# Predicting Climate Change Effects on the Potential Distribution of Two Invasive Cryptic Species of the *Bemisia tabaci* Species Complex in China

**DOI:** 10.3390/insects13121081

**Published:** 2022-11-23

**Authors:** Yantao Xue, Congtian Lin, Yaozhuo Wang, Wanxue Liu, Fanghao Wan, Yibo Zhang, Liqiang Ji

**Affiliations:** 1 Key Laboratory of Animal Ecology and Conservation Biology, Institute of Zoology, Chinese Academy of Sciences, Beijing 100101, China; 2 State Key Laboratory for Biology of Plant Diseases and Insect Pests, Institute of Plant Protection, Chinese Academy of Agricultural Sciences, Beijing 100193, China; 3 University of Chinese Academy of Sciences, Beijing 100049, China; 4 National Basic Science Data Center, Beijing 100190, China; 5 Key Laboratory of Zoological Systematics and Application of Hebei Province, College of Life Sciences, Hebei University, Baoding 071002, China

**Keywords:** whitefly, invasive species, biological invasion, MaxEnt, climate change

## Abstract

**Simple Summary:**

Biological invasions have become an ecological issue worldwide, and climate change may further influence the spread of invasive species. In this study, the latest Coupled Model Intercomparison Project Phase 6 (CMIP6) data were used to predict the suitable habitats of two invasive cryptic species, namely Middle East-Asia Minor 1 (MEAM1) and Mediterranean (MED), of the *Bemisia tabaci* species complex in China based on a species distribution modeling technique in current and future climate change scenarios from the present to the end of the 21st century. The results demonstrated differences in the future changes and dispersal in their suitable habitats in China under different future climatic scenarios, which suggests that climate change would have greater but divergent impacts on the potential distribution of these two invasive cryptic species. It has direct implications for developing regional management strategies in China for certain periods in the future and also provides a practical approach that can be applied to other invasive species.

**Abstract:**

Middle East-Asia Minor 1 (MEAM1) and Mediterranean (MED) are two invasive cryptic species of the *Bemisia tabaci* species complex (Hemiptera: Aleyrodidae) that cause serious damage to agricultural and horticultural crops worldwide. To explore the possible impact of climate change on their distribution, the maximum entropy (MaxEnt) model was used to predict the potential distribution ranges of MEAM1 and MED in China under current and four future climate scenarios, using shared socioeconomic pathways (SSPs), namely SSP1-2.6, SSP2-4.5, SSP3-7.0, and SSP5-8.5, over four time periods (2021–2040, 2041–2060, 2061–2080, and 2081–2100). The distribution ranges of MEAM1 and MED were extensive and similar in China under current climatic conditions, while their moderately and highly suitable habitat ranges differed. Under future climate scenarios, the areas of suitable habitat of different levels for MEAM1 and MED were predicted to increase to different degrees. However, the predicted expansion of suitable habitats varied between them, suggesting that these invasive cryptic species respond differently to climate change. Our results illustrate the difference in the effects of climate change on the geographical distribution of different cryptic species of *B. tabaci* and provide insightful information for further forecasting and managing the two invasive cryptic species in China.

## 1. Introduction

Invasive alien species (IAS) pose major threats to the ecological environment, biodiversity, human health, agriculture, and forestry directly and/or indirectly [1,2]. In recent years, biological invasions have become a hot issue worldwide in the context of increasing global trade [3]. It is estimated that global economic costs of biological invasions have been responsible for at least USD 1.288 trillion over the past 50 years, and in 2017 alone, cost up to USD 162.7 billion [4]. The United Nations’ Sustainable Development Goals (SDGs) have targeted IAS to prevent their introduction and reduce their impact [5]. Climate change can affect various aspects of IAS, including introduction pathways and potential survival areas, often resulting in increased risk to invaded ecosystems [6,7,8,9]. Thus, exploring interactions between climate change and biological invasions has become an urgent issue [10,11].

Two invasive cryptic species of the *Bemisia tabaci* species complex (Gennadius, 1889) (Hemiptera: Aleyrodidae), namely Middle East-Asia Minor 1 (MEAM1) and Mediterranean (MED), also commonly known as *B. tabaci* biotype B and Q, respectively, are destructive and polyphagous IAS of vegetable and ornamental crops worldwide [12]. They affect many plant species not only by feeding on phloem and excreting honeydew but also by transmitting hundreds of plant viruses [12]. *B. tabaci* cryptic species are particularly destructive in tropical and subtropical regions of the world where environmental conditions favor their growth and population buildup [13,14,15]. These two invasive cryptic species were first detected in China in the middle to late 1990s and early 21st century, respectively, and since then, they have spread rapidly across China and replaced the native whiteflies, causing great damage to agricultural production [16,17].

The distribution patterns and dynamics of MEAM1 and MED in China have been studied widely through field surveys [18,19,20,21]. Recent field surveys have revealed that MED is gradually displacing the earlier invader MEAM1, resulting in MED being the dominant cryptic species in many invaded regions in China [22]. It has been proposed that this displacement can be attributed to hosting suitability and feeding behavior [23], reproductive interference [24], insecticide resistance [25], the tolerance of MED to high temperatures [26], and its genetic diversity by multiple invasions [27], as well as the differences in their ecological niches and adaptations to the environment [28]. Despite morphological indistinguishability, these two invasive cryptic species exhibit significant differences in many aspects including virus transmission [29,30,31,32]. Therefore, it can be hypothesized that there may be differences in their response to climate change. In addition, the different sensitivities of these two invasive cryptic species to predominant parasitoids have also been evidenced [33]. Clarifying the differences in the effects of future climate change on their potential distribution will not only help further understand their difference in ecological characteristics but may also contribute to the formulation of effective control measures.

The maximum entropy (MaxEnt) model, an accepted specific algorithm of species distribution models (SDMs), has been widely used to predict the potential geographic distribution and habitat suitability of species, including IAS, under different climate scenarios [34,35]. MaxEnt is a general-purpose method based on machine learning and the maximum entropy principle. It uses species’ presence-only records and relevant environmental variables to express the suitability of the species for each grid cell and thus predict its potential distribution in the entire study area [35,36]. The method is simple to operate, has a short model-running time and small sample size requirements, and has been shown to perform better than other methods [36]. Previous studies used this method to effectively predict the current and/or future distribution of the *B. tabaci* species complex in different regions of the world [37,38,39,40]. In China, Ren et al. [41] and Zhao et al. [42] predicted the current potential distribution of the *B. tabaci* species complex as a species rather than for different cryptic species. Considering the above-mentioned differences among *B. tabaci* cryptic species, it is necessary to predict the suitable habitat for different cryptic species, especially for the two most invasive members of the *B. tabaci* species complex [28]. Meanwhile, there remains a dearth of information on their respective potential distribution, especially regarding predictions of future distribution trends under climate change scenarios.

In the present study, the suitable habitat ranges of MEAM1 and MED, two distinct invasive cryptic species of the *B. tabaci* species complex, were separately predicted in China under current and different future climate scenarios using the MaxEnt model based on the open field occurrence data obtained from extensive field surveys and the literature, as well as high-resolution environmental variables. The potential changes in their habitat range and dispersal trends under predicted future climate scenarios from the present to the end of the 21st century were also investigated. This study aimed to identify the differences in the response of MEAM1 and MED to future climate change and provide a reference for forecasting and managing these invasive pests in China.

## 2. Materials and Methods

### 2.1. Occurrence Data

**Figure 1:**
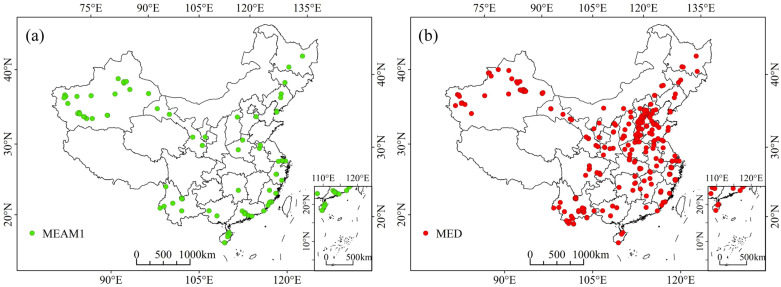
Distribution of the occurrence records used in the MaxEnt models of (**a**) MEAM1 and (**b**) MED cryptic species of the *Bemisia tabaci* species complex in China.

The open field occurrence data of MEAM1 and MED in China were obtained from two sources. First, field surveys of adult whiteflies were carried out in open fields across China from 2016 to 2018. All individuals were identified as either cryptic species of the *B. tabaci* species complex or other whitefly species according to De Barro et al. [12], and only the results for the cryptic species of MEAM1 and MED were retained. Second, their occurrence data from the scientific literature that provided detailed information on the coordinates in the wild in China were adopted after being checked [43,44,45]. To reduce sampling deviation, only one occurrence record was kept in each grid cell to match the spatial resolution of the environmental variable data below. After filtering, 82 and 270 occurrence records of MEAM1 and MED, respectively, remained for modeling (Figure 1; Table S1).

### 2.2. Environmental Variables

Twenty environmental variables related to the distribution of the *B. tabaci* species complex were considered for developing the MaxEnt model (Table 1). This included 19 bioclimatic variables (bio1–bio19) and 1 topographic variable (elevation), with a spatial resolution of 2.5 arcmin (~5 km at the equator) that were obtained from the WorldClim Global Climate Database (version 2.1, http://www.worldclim.org, accessed on 25 March 2021) [46]. These variables were transformed to ASCII format and then extracted from the base map of China in ArcGIS 10.6 (Esri, Redlands, CA, USA). To eliminate possible multicollinearity between environmental variables, which might affect the accuracy of prediction and reduce the performance of the models [47], the Pearson correlation coefficients (*r*) of these variables for the current period (1970–2000) were calculated using the Spatial Analyst tools in the ArcToolbox of ArcGIS 10.6 (Table S2). If two variables were highly correlated (|*r*| ≥ 0.85), then only one variable was retained in the final models [28]. Accordingly, annual mean temperature (bio1), mean diurnal range (bio2), isothermality (bio3), annual temperature range (bio7), annual precipitation (bio12), precipitation seasonality (bio15), precipitation of the coldest quarter (bio19), and elevation (elev) were retained for modeling.

To predict the future potential distributions of MEAM1 and MED, we used the four latest climate change scenarios of bioclimatic variables, that is, shared socioeconomic pathways (SSPs): SSP1-2.6, SSP2-4.5, SSP3-7.0, and SSP5-8.5, from the Coupled Model Intercomparison Project Phase 6 (CMIP6). These SSPs represent continually increasing concentrations of greenhouse gas (GHG) emissions that are updated by the Representative Concentration Pathways (RCPs) in CMIP5 [48,49]. The SSPs data projected by the Beijing Climate Center Climate System Model with Moderate Resolution (BCC-CSM2-MR) was selected for the present study. Its performance and functions have been effectively improved after more than 10 years of development and thus better reflect the magnitude of future climate change in China compared with other models [50]. It has been widely used to predict the future distribution of species in China. Four future periods were included in each SSP, namely 2030s (average for 2021–2040), 2050s (average for 2041–2060), 2070s (average for 2061–2080), and 2090s (average for 2081–2100). The same elevation data were used in different scenarios and periods.

### 2.3. Model Development

MaxEnt software (version 3.4.4, http://biodiversityinformatics.amnh.org/open_source/maxent/, accessed on 18 September 2020) [35] was used to predict the potential distribution of MEAM1 and MED in China under current and future climate scenarios. The models were set to 10-fold using the cross-validation method. The maximum number of iterations was increased from 500 (default) to 5000 to ensure convergence. The “fade-by-clamping” option was selected to eliminate extrapolations outside the environmental range [51]. The “cloglog” output format was checked for model analysis.

The ENMeval package [52] was used to optimize the models to avoid overfitting by adjusting the feature combination (FC) and regularization multiplier (RM) in R software (version 4.0.5, https://www.r-project.org/, accessed on 1 April 2021). MaxEnt provides five features, namely linear (L), quadratic (Q), hinge (H), product (P), and threshold (T) [36]. Six different FCs (L, LQ, H, LQH, LQHP, and LQPHT) were tested with RM ranging from 0.5 to 4.0 in increments of 0.5. The candidate models with the lowest delta value of the Akaike information criterion coefficient (AICc) were selected for each cryptic species. The ENMeval results revealed that the optimal parameters for FC and RM were LQ and 0.5 in the final models for both cryptic species (Figure S1).

### 2.4. Model Evaluation

The performance of the MaxEnt models was evaluated using the threshold-independent Receiver Operating Characteristic (ROC) curve, and the area under the curve (AUC) value was positively correlated with the performance of the prediction model, whereby the higher the AUC value, the higher the reliability [53]. In general, the model performance was classified, depending on the AUC value, as failed (0.5–0.6), poor (0.6–0.7), general (0.7–0.8), good (0.8–0.9), and excellent (0.9–1.0) [35]. The results of the model evaluation showed that the MaxEnt models for the two cryptic species had high predictive abilities, with average AUC values of 0.879 for MEAM1 and 0.870 for MED (Figure S2). This means that the prediction results generated using the MaxEnt model are reliable.

### 2.5. Habitat Suitability Classification

The MaxEnt prediction results generated based on the value of the presence probability of MEAM1 and MED were imported into ArcGIS 10.6 for suitability classification and visual interpretation. The values of each grid cell range from 0 to 1, with higher values indicating a higher presence probability of species. We used the maximum test sensitivity plus specificity (MTSPS) threshold to determine the suitable and unsuitable habitats. Therefore, four levels of habitat suitability were defined for MEAM1 and MED accordingly: unsuitable (0–MTSPS), slightly suitable (MTSPS–0.5), moderately suitable (0.5–0.7), and highly suitable (0.7–1) [54].

### 2.6. Distribution Change Estimation

We compared current and future distributions of MEAM1 and MED using a variety of different methods to determine the response of different cryptic species to climate change. First, the potential distribution areas of different habitat suitability for the two cryptic species under different climate scenarios were calculated using the zonal statistics tool in ArcGIS 10.6. Then, the suitable areas in each period under four future climate scenarios were compared with the current areas to calculate the extent of habitat change (expansion, no change, or contraction) based on binary models, i.e., the distribution maps of suitable and unsuitable habitats, using the distribution changes between binary SDMs tool in the SDM toolbox [55]. Additionally, the distributional centroid shifts of the predicted distribution in different periods under four future climate scenarios were measured separately using the centroid changes (Lines) tool in the SDM toolbox [55] in ArcGIS 10.6 to demonstrate the different directions and distances of distribution area shifts of the two cryptic species.

## 3. Results

### 3.1. Predicted Current Potential Distribution

**Figure 2:**
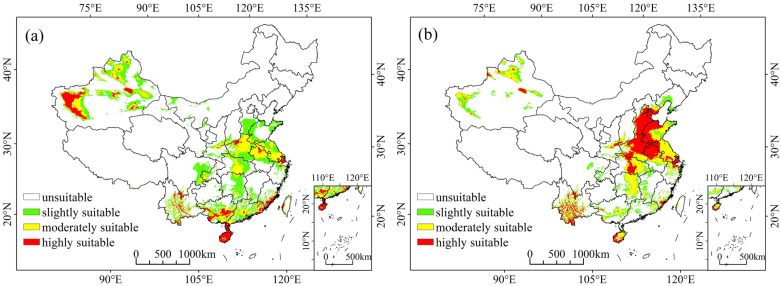
Suitable habitat distribution of (**a**) MEAM1 and (**b**) MED cryptic species of the *Bemisia tabaci* species complex in China under current climatic conditions.

Based on distribution records and present-time environmental variables, the modeling results produced by MaxEnt show the potential distribution with different habitat suitability for MEAM1 and MED under the current climate conditions in China (Figure 2). These two cryptic species are widespread in China and have overlapping areas in many provinces. Among them, MEAM1 is mainly distributed in the northern, central, eastern, and southern parts of China and Xinjiang, with broken distributions in southwest China. Its highly suitable habitats are mainly concentrated in south China and Xinjiang, with sporadic distribution in a few parts of central, eastern, and southwest China (Figure 2a). The current distribution of MED is similar to that of MEAM1 except in parts of north China and Xinjiang. Its highly suitable habitats, which differ from those of MEAM1, are mainly concentrated in north China and the northern parts of eastern and central China, as well as sporadically distributed in certain parts of south and southwest China and Xinjiang (Figure 2b). The suitable distribution area of MEAM1 is 188.67 × 10^4^ km^2^ (accounting for 19.62% of the total land area of China), with slightly, moderately, and highly suitable areas of 101.66 × 10^4^ km^2^ (10.60%), 60.84 × 10^4^ km^2^ (6.33%), and 26.18 × 10^4^ km^2^ (2.72%), respectively (Table S3). For MED, its suitable distribution area is 153.99 × 10^4^ km^2^ (16.01%), with slightly, moderately, and highly suitable areas of 44.67 × 104 km^2^ (4.64%), 58.27 × 10^4^ km^2^ (6.06%), and 51.00 × 10^4^ km^2^ (5.31%), respectively (Table S3).

### 3.2. Predicted Future Potential Distribution

**Figure 3:**
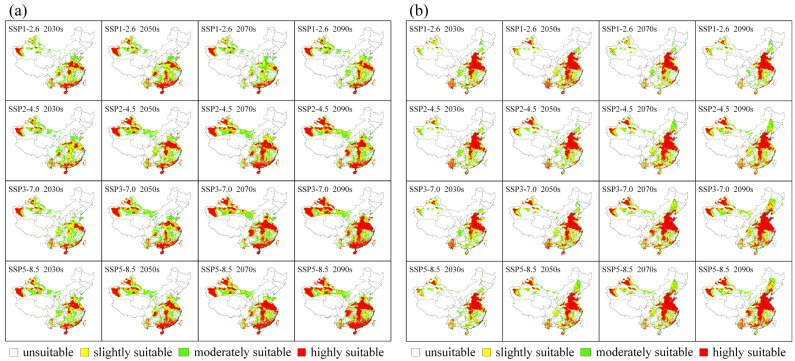
Suitable habitat distribution of (**a**) MEAM1 and (**b**) MED cryptic species of the *Bemisia tabaci* species complex in China under future climate scenarios. SSP, shared socioeconomic pathway.

A global climate model more suitable for China and its four SSPs data were used to predict the potential distribution of MEAM1 and MED in China under future climate conditions in 2030s, 2050s, 2070s, and 2090s (Figure 3). Habitat suitability for MEAM1 will continually increase based on its current distribution mainly in northwest China. Many of its slightly suitable habitats under current climate conditions will gradually become moderately or highly suitable and tend to expand into the surrounding areas over time (Figure 3a). MED will expand its suitable habitats mainly in the northeast and northwest of China. Its highly suitable habitats will tend to expand in north China, south China, and Xinjiang, while the moderately suitable habitats will expand in northeast, south, southwest China, and Xinjiang (Figure 3b).

**Figure 4:**
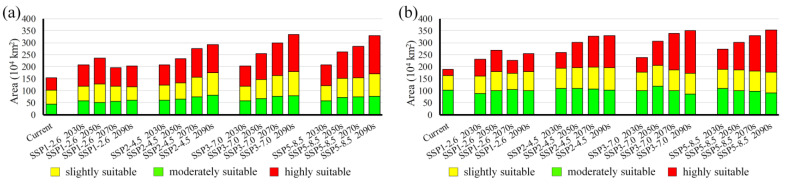
Comparison of potential suitable areas for (**a**) MEAM1 and (**b**) MED cryptic species of the *Bemisia tabaci* species complex in China under current and future climate scenarios. SSP, shared socioeconomic pathway.

For both MEAM1 and MED, the total suitable areas will continuously increase under all future climate scenarios except in the 2070s and the 2090s under the SSP1-2.6 scenarios, although both will still increase relative to the current status (Figure 4; Table S3). Under all future climate scenarios, the suitable areas for MEAM1 and MED will increase by 20.00–87.16% and 27.93–116.88%, respectively, compared with their current distribution areas. The maximum suitable area for MEAM1 will occur in the 2090s under the SSP5-8.5 scenario (353.13 × 10^4^ km^2^), and that for MED will occur in the 2090s under the SSP3-7.0 scenario (333.96 × 10^4^ km^2^) (Table S3). For highly suitable habitats, the area for MEAM1 can be up to 179.52 × 10^4^ km^2^ (in the 2090s under the SSP3-7.0 scenario), representing an increase of 5.86 times compared with the current status. The area for MED can be up to 159.38 × 10^4^ km^2^ (in the 2090s under the SSP5-8.5 scenario), which is 2.12 times higher than the current status (Table S3).

### 3.3. Habitat Changes under Future Climate Scenarios

**Figure 5:**
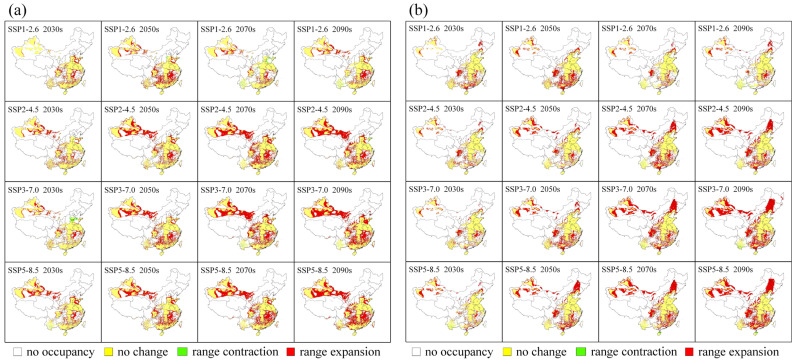
Predicted habitat changes for (**a**) MEAM1 and (**b**) MED cryptic species of the *Bemisia tabaci* species complex in China under future climate scenarios compared with the current status. SSP, shared socioeconomic pathway.

The expansion and contraction of suitable habitats for MEAM1 and MED are intuitive in the result of binary SDMs (Figure 5), which highlights their differences in the suitable habitat changes under different future climate scenarios. The suitable habitats of both cryptic species are predicted to significantly expand in the future. The newly suitable areas for MEAM1 will mainly appear in the south, east, and northwest of China, and there will be a small contraction in parts of southwest and east China in the 2070s and the 2090s under the SSP1-2.6 scenario and the 2030s under the SSP3-7.0 scenario (Figure 5a). The suitable habitats for MED are predicted to expand mainly in northeast, south, and southwest China and Xinjiang, and sporadically in the north, central, and east China (Figure 5b). Furthermore, the predicted suitable habitats for MED will likely see small contractions in southwest and south China under some future scenarios (Figure 5b).

### 3.4. Centroid Shifts between Current and Future Distribution

**Figure 6:**
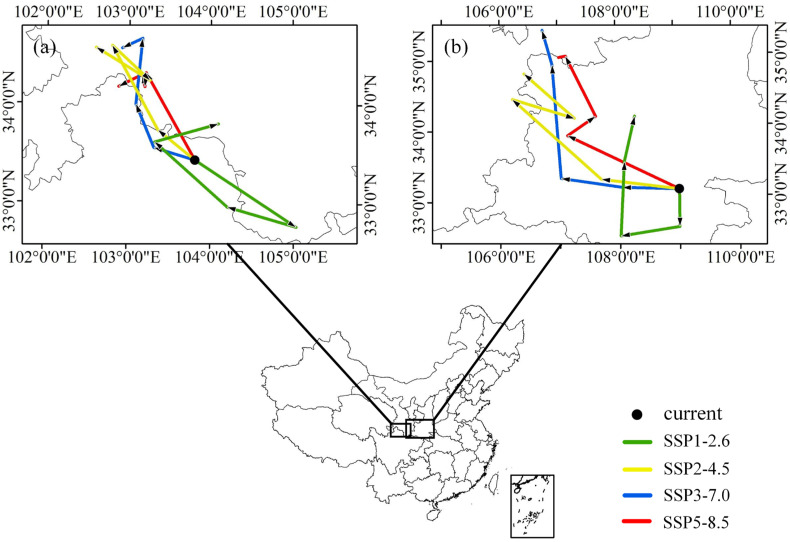
Distributional centroid shifts of suitable areas for (**a**) MEAM1 and (**b**) MED cryptic species of the *Bemisia tabaci* species complex in China in different periods (2030s, 2050s, 2070s, and 2090s) under four future climate change scenarios. SSP, shared socioeconomic pathway.

The distributional centroid shifts of suitable habitats for MEAM1 and MED were measured to reflect the directions of predicted distribution changes over time, further illustrating their responses to future climate change (Figure 6). The current distributional centroids of MEAM1 and MED in China were located in northeast Sichuan and southeast Shaanxi, respectively (Figure 6a). Under the SSP1-2.6 scenario, the distributional centroids of these two cryptic species moved around their current centroids without a consistent direction over time. Under the other three future climate scenarios (SSP2-2.4, SSP3-7.1, and SSP5-8.5), there appeared to be a similarity in the simulated changes of the distributional centroids of these two cryptic species, with both moving roughly north-westward overall over time (Figure 6b). However, each period’s migration direction and distance differed (Table S4).

## 4. Discussion

Predicting the potential distribution of IAS under climate change scenarios provides important references for risk analysis, early detection, and prevention or control-management [56]. As important tools for biogeographic research, SDMs are now increasingly used to predict changes in the distribution of IAS in the context of global warming and increasing trade [57,58]. In the present study, MaxEnt models were constructed for two invasive cryptic species of the *B. tabaci* species complex, namely MEAM1 and MED, according to their occurrence data and multiple environmental variables under current and predicted future conditions. To the best of our knowledge, this is the first study to independently predict the potential patterns in suitable habitats for the two invasive cryptic species in China under both current and future climate scenarios. Thus, the results are important for furthering our understanding of these pests in China and improving our ability to control and manage them.

In the current models, MEAM1 and MED have extensive and similar distribution ranges in China, which is generally consistent with the results of field surveys [22]. Previous studies have shown that the *B. tabaci* species complex is predicted to be distributed mainly in southern, eastern, and northern China under the current climate conditions using the MaxEnt model [41,42]. This seems to be an extended superposition of the two cryptic species’ results of the present study. It is because these studies were conducted with the entire *B. tabaci* species complex, while the present study dealt with each invasive cryptic species independently. Additionally, more and broader distribution data were used to construct the model. Furthermore, the difference in distribution between MEAM1 and MED is mainly reflected in the suitability of the habitats; that is, the distribution patterns of slight, moderate, and high suitability habitats. This may indicate that despite similarities in the environmental requirements for their survival, there may be subtle differences in their adaptation ability and ecological niches [28].

In the future models, both the total suitable habitats and high-suitability habitats of these two cryptic species are projected to significantly increase under all future climatic scenarios compared with the current scenario. However, the expansion of suitable habitats of MEAM1 and MED in northern China is markedly different under climate change scenarios. The temperature in northeast China is rising faster than that in other regions under global warming [59]. These two cryptic species have different tolerance capacities for unfavorable temperatures [26], which may explain this phenomenon to some extent. Ramos et al. [39] predicted the global suitability of MEAM1 and MED for open field tomato cultivation under future climate change conditions using the MaxEnt model. Their results were notably missing many suitable habitats in China predicted in the present study, such as northeast China, northwest China, and Xinjiang regions. Using more recent occurrence data collected extensively from open fields across China and modeling the two invasive cryptic species separately in this study may be the main reason for this discrepancy. Thus, the results of the present study may be more accurate as far as China is concerned. However, our results are consistent with the previous studies in that their future distribution in China, as well as other regions of the world, will gradually increase [37,38,39,60]. In addition to the expansion in suitable habitat areas, our results also indicate significant changes in the suitability of different regions of China for the two invasive cryptic species under future climatic scenarios, which also implies a change in the damage levels these pests may cause in the future.

From the present to the end of the 21st century, the shifts in potentially suitable areas and the migration paths of the distributional centroids of both MEAM1 and MED show significant complexity and variability under different future climate scenarios. This suggests that differences in the intensity of global warming can significantly affect the distribution and possible dispersal routes of invasive cryptic species of the *B. tabaci* species complex in China. As GHG emissions increase in the future, the risk of invasive cryptic species appears to become increasingly serious. Notably, according to the results of this study, MEAM1 and MED will never extend onto the Qinghai–Tibet Plateau even with the substantial expansion of their suitable habitats under high GHG emission scenarios. In recent field surveys, only some whitefly species identified as MED cryptic species were found in greenhouses in Tibet [61,62]. The probability of survival of the *B. tabaci* species complex was higher at low elevations and decreased as the elevation increased [28,42]. Additionally, in the United States, except for two field reports, MED has been mostly restricted to greenhouse conditions [63]. These studies suggest that elevation and other unknown natural factors may be an important limiting factor for these two cryptic species’ survival and natural dispersal in the open field. Nevertheless, our results indicate that both invasive cryptic species still pose a serious threat to agricultural production in China, whether currently or in the future.

As with the invasive cryptic species of the *B. tabaci* species complex, the subject of this study, other IAS, including plants and insects, have also been predicted to increase potential risk to China’s native flora and fauna under climate change conditions [10,64,65]. Most will expand northward or to higher latitudes. The negative impact of climate change on invasive alien species, such as bullfrogs *Lithobates catesbeianus*, an invasive amphibian species in China, were predicted in previous studies [66]. Taken together, it can be concluded with certain confidence that different IAS may have varied responses to climate change. This further justifies the need for separate predictions of different invasive cryptic species of the *B. tabaci* species complex.

Despite its advantages and high accuracy, the MaxEnt model only established the relationship between the species’ adaptive distribution and specific environmental factors [35,36]. However, the distribution of species is not only influenced by environmental factors, but also related to interspecific interactions, host distribution, and various other factors, and the distribution and spread of IAS, in particular, are also affected by human activities [11]. In addition, the adaptive capacity of insects to climate change has also been evidenced and suggested to be incorporated into SDMs [67,68]. However, these data are difficult to obtain or predict, especially for future scenarios. In this study, therefore, the projection results were only obtained by considering climate change as a factor, which still has important guiding significance for predicting and managing these invasive pests. Future studies could consider the influence of more relevant environmental factors and human activities on predicting the future geographic distribution of MEAM1 and MED, based on more data.

## Figures and Tables

**Table 1:** Environmental variables considered in the MaxEnt models of MEAM1 and MED cryptic species of the *Bemisia tabaci* species complex in China.

Variable Type	Variable Code	Description
Bioclimatic variables	bio1	Annual mean temperature
	bio2	Mean diurnal range (mean of monthly (max temp.−min temp.))
	bio3	Isothermality (bio2/bio7) (×100)
	bio4	Temperature seasonality (standard deviation ×100)
	bio5	Maximum temperature of the warmest month
	bio6	Minimum temperature of the coldest month
	bio7	Temperature annual range (bio5−bio6)
	bio8	Mean temperature of the wettest quarter
	bio9	Mean temperature of the driest quarter
	bio10	Mean temperature of the warmest quarter
	bio11	Mean temperature of the coldest quarter
	bio12	Annual precipitation
	bio13	Precipitation of the wettest month
	bio14	Precipitation of the driest month
	bio15	Precipitation seasonality (coefficient of variation)
	bio16	Precipitation of the wettest quarter
	bio17	Precipitation of the driest quarter
	bio18	Precipitation of the warmest quarter
	bio19	Precipitation of the coldest quarter
Elevation	elev	Ground height above sea level

## Data Availability

The data presented in this study are available in the article.

## Supplementary Materials

The following supporting information can be downloaded at: https://www.mdpi.com/article/10.3390/insects13121081/s1: Figure S1: ENMeval output for (a) MEAM1 and (b) MED cryptic species of the *Bemisia tabaci* species complex, showing that the optimal parameter combination of feature class and regularization multiplier in the MaxEnt models was LQ and 0.5, respectively, for both of them; Figure S2: Receiver operating characteristic curve verification of the prediction for (a) MEAM1 and (b) MED cryptic species of the *Bemisia tabaci* species complex; Table S1: Detailed information for the occurrence records used in the MaxEnt models of MEAM1 and MED cryptic species of the *Bemisia tabaci* species complex in China; Table S2: The correlation matrix of environmental variables; Table S3: Potential suitable areas for MEAM1 and MED of the *Bemisia tabaci* species complex in China under current and future climate scenarios; Table S4: The geographic coordinates of the distributional centroid of MEAM1 and MED cryptic species of the *Bemisia tabaci* species complex in China under current and future climate scenarios, and their migration distance relative to the previous period.
