# Supplementary material for: Predicting Climate Change Effects on the Potential Distribution of Two Invasive Cryptic Species of the Bemisia tabaci Species Complex in China
Source: Insects. 2022 Nov 23;13(12):1081. doi: 10.3390/insects13121081 (PMC9783486; doi:10.3390/insects13121081)
Supplement: Supplementary file 1 [file insects-13-01081-s001.zip › insects-2038294-supplementary.pdf]

Supplementary Materials

Table S1. Detailed information for the occurrence records used in the MaxEnt models of MEAM1 and MED cryptic species of the *Bemisia tabaci* species complex in China.

Cryptic species	Place of collection (Location, province)	Latitude	Longitude	Time of collection	Source [1]
MEAM1	Xiqing, Tianjin	38°53' N	117°16' E	Aug. 2017	Field surveys
MEAM1	Xinzhou, Shanxi	39°11' N	113°15' E	Jun. 2016	Field surveys
MEAM1	Shenyang, Liaoning	41°51' N	123°25' E	Aug. 2016	Field surveys
MEAM1	Dalian, Liaoning	39°14' N	121°43' E	Sep. 2016	Field surveys
MEAM1	Dalian, Liaoning	39°06' N	121°42' E	Aug. 2017	Field surveys
MEAM1	Liaoyang, Liaoning	41°16' N	123°09' E	Aug. 2016	Field surveys
MEAM1	Siping, Jilin	43°30' N	124°48' E	Aug. 2017	Field surveys
MEAM1	Harbin, Heilongjiang	45°45' N	126°35' E	Aug. 2017	Field surveys
MEAM1	Jiamusi, Heilongjiang	46°48' N	130°22' E	Aug. 2017	Field surveys
MEAM1	Qingpu, Shanghai	31°06' N	121°08' E	Sep. 2017	Field surveys
MEAM1	Pudong, Shanghai	30°59' N	121°46' E	Aug. 2016	Field surveys
MEAM1	Xuzhou, Jiangsu	34°07' N	117°21' E	Aug. 2016	Field surveys
MEAM1	Suzhou, Jiangsu	31°05' N	120°26' E	Sep. 2017	Field surveys
MEAM1	Wenzhou, Zhejiang	27°57' N	120°34' E	Aug. 2016	Field surveys
MEAM1	Jinhua, Zhejiang	29°03' N	119°45' E	Aug. 2016	Field surveys
MEAM1	Jinhua, Zhejiang	29°02' N	119°40' E	Aug. 2017	Field surveys
MEAM1	Suzhou, Anhui	33°37' N	117°06' E	Aug. 2016	Field surveys
MEAM1	Fuzhou, Fujian	25°56' N	119°15' E	Aug. 2016	Field surveys
MEAM1	Fuzhou, Fujian	25°58' N	119°13' E	Aug. 2017	Field surveys
MEAM1	Xiamen, Fujian	24°39' N	118°18' E	Aug. 2016	Field surveys
MEAM1	Xiamen, Fujian	24°38' N	118°02' E	Aug. 2016	Field surveys
MEAM1	Xiamen, Fujian	24°37' N	118°14' E	Aug. 2017	Field surveys
MEAM1	Xiamen, Fujian	24°35' N	117°54' E	Jul. 2018	Field surveys
MEAM1	Zhangzhou, Fujian	24°07' N	117°36' E	Jul. 2018	Field surveys
MEAM1	Nanping, Fujian	26°33' N	118°07' E	Aug. 2016	Field surveys
MEAM1	Luoyang, Henan	33°44' N	112°53' E	Aug. 2016	Field surveys
MEAM1	Xinxiang, Henan	35°17' N	113°56' E	Aug. 2016	Field surveys
MEAM1	Hengyang, Hunan	26°58' N	112°22' E	Aug. 2016	Field surveys
MEAM1	Guangzhou, Guangdong	23°09' N	113°23' E	May. 2016	Field surveys
MEAM1	Guangzhou, Guangdong	23°09' N	113°21' E	Jul. 2016	Field surveys
MEAM1	Guangzhou, Guangdong	23°00' N	113°16' E	Dec. 2016	Field surveys
MEAM1	Shenzhen, Guangdong	22°42' N	113°55' E	Jul. 2016	Field surveys
MEAM1	Shenzhen, Guangdong	22°31' N	114°31' E	Jul. 2018	Field surveys
MEAM1	Shantou, Guangdong	23°16' N	116°44' E	Jul. 2018	Field surveys
MEAM1	Foshan, Guangdong	23°30' N	112°51' E	Jun. 2016	Field surveys
MEAM1	Nanning, Guangxi	22°51' N	108°15' E	Jun. 2016	Field surveys
MEAM1	Baise, Guangxi	23°43' N	106°48' E	Mar. 2016	Field surveys
MEAM1	Haikou, Hainan	20°00' N	110°20' E	Jul. 2016	Field surveys

MEAM1	Haikou, Hainan	19°40' N	110°22' E	Sep. 2018	Field surveys
MEAM1	Sanya, Hainan	18°18' N	109°32' E	May. 2016	Field surveys
MEAM1	Sanya, Hainan	18°19' N	109°29' E	Feb. 2018	Field surveys
MEAM1	Tunchang, Hainan	19°17' N	110°05' E	Apr. 2018	Field surveys
MEAM1	Chengmai, Hainan	19°44' N	110°08' E	Aug. 2016	Field surveys
MEAM1	Yuxi, Yunnan	23°41' N	101°49' E	May. 2012	Hu et al. 2014
MEAM1	Lincang, Yunnan	24°55' N	100°08' E	Aug. 2012	Hu et al. 2014
MEAM1	Chuxiong, Yunnan	25°49' N	101°44' E	Sep. 2016	Hu et al. 2018
MEAM1	Chuxiong, Yunnan	25°51' N	101°46' E	Sep. 2016	Hu et al. 2018
MEAM1	Chuxiong, Yunnan	25°42' N	101°53' E	Sep. 2016	Hu et al. 2018
MEAM1	Chuxiong, Yunnan	25°46' N	101°51' E	Sep. 2016	Hu et al. 2018
MEAM1	Chuxiong, Yunnan	25°43' N	101°46' E	Nov. 2014	Hu et al. 2018
MEAM1	Dehong, Yunnan	24°01' N	97°51' E	Oct. 2010	Hu et al. 2014
MEAM1	Dehong, Yunnan	24°21' N	98°34' E	Oct. 2010	Hu et al. 2014
MEAM1	Nujiang, Yunnan	27°39' N	98°43' E	Aug. 2016	Hu et al. 2018
MEAM1	Lanzhou, Gansu	36°06' N	103°41' E	Aug. 2018	Field surveys
MEAM1	Tianshui, Gansu	34°42' N	105°40' E	Aug. 2016	Field surveys
MEAM1	Jiuquan, Gansu	40°31' N	95°47' E	Aug. 2016	Field surveys
MEAM1	Jiuquan, Gansu	39°45' N	98°32' E	Aug. 2016	Field surveys
MEAM1	Guyuan, Ningxia	36°06' N	106°15' E	Aug. 2017	Field surveys
MEAM1	Urumqi, Xinjiang	43°49' N	87°34' E	Aug. 2016	Field surveys
MEAM1	Urumqi, Xinjiang	44°01' N	87°26' E	Aug. 2016	Field surveys
MEAM1	Urumqi, Xinjiang	43°58' N	87°25' E	Jul. 2017	Field surveys
MEAM1	Tulufan, Xinjiang	42°59' N	89°09' E	Jun. 2015	Jia et al. 2021
MEAM1	Hami, Xinjiang	42°50' N	93°27' E	Apr. 2016	Jia et al. 2021
MEAM1	Changji, Xinjiang	44°09' N	88°01' E	Mar. 2017	Jia et al. 2021
MEAM1	Bayingol, Xinjiang	38°07' N	85°34' E	Aug. 2016	Jia et al. 2021
MEAM1	Bayingol, Xinjiang	38°08' N	85°30' E	Aug. 2016	Jia et al. 2021
MEAM1	Bayingol, Xinjiang	41°46' N	86°08' E	Mar. 2016	Jia et al. 2021
MEAM1	Kashi, Xinjiang	38°17' N	77°11' E	Aug. 2016	Jia et al. 2021
MEAM1	Kashi, Xinjiang	39°24' N	76°05' E	Sep. 2015	Jia et al. 2021
MEAM1	Kashi, Xinjiang	39°14' N	76°22' E	Sep. 2015	Jia et al. 2021
MEAM1	Kashi, Xinjiang	38°55' N	76°08' E	Sep. 2015	Jia et al. 2021
MEAM1	Kashi, Xinjiang	39°47' N	78°31' E	Sep. 2015	Jia et al. 2021
MEAM1	Hetian, Xinjiang	37°27' N	79°52' E	Jun. 2015	Jia et al. 2021
MEAM1	Hetian, Xinjiang	37°04' N	82°42' E	Aug. 2016	Jia et al. 2021
MEAM1	Hetian, Xinjiang	36°50' N	81°38' E	Aug. 2016	Jia et al. 2021
MEAM1	Hetian, Xinjiang	36°59' N	81°04' E	Aug. 2016	Jia et al. 2021
MEAM1	Hetian, Xinjiang	36°59' N	80°58' E	Aug. 2016	Jia et al. 2021
MEAM1	Hetian, Xinjiang	37°14' N	79°41' E	Aug. 2016	Jia et al. 2021
MEAM1	Hetian, Xinjiang	37°17' N	79°39' E	Aug. 2016	Jia et al. 2021
MEAM1	Hetian, Xinjiang	37°15' N	80°05' E	Apr. 2016	Jia et al. 2021
MEAM1	Shihezi, Xinjiang	44°19' N	86°03' E	Aug. 2016	Field surveys
MEAM1	Alaer, Xinjiang	40°32' N	81°17' E	Mar. 2017	Jia et al. 2021

MED	Tongzhou, Beijing	39°47' N	116°45' E	Aug. 2017	Field surveys
MED	Changping, Beijing	40°09' N	116°13' E	Jul. 2017	Field surveys
MED	Daxing, Beijing	39°38' N	116°19' E	Jul. 2017	Field surveys
MED	Shunyi, Beijing	40°01' N	116°46' E	Aug. 2017	Field surveys
MED	Haidian, Beijing	40°06' N	116°13' E	Aug. 2017	Field surveys
MED	Haidian, Beijing	40°01' N	116°16' E	Oct. 2018	Field surveys
MED	Beichen, Tianjin	39°13' N	117°07' E	Aug. 2017	Field surveys
MED	Wuqing, Tianjin	39°31' N	116°58' E	Aug. 2017	Field surveys
MED	Xiqing, Tianjin	38°53' N	117°16' E	Aug. 2017	Field surveys
MED	Dongli, Tianjin	39°03' N	117°22' E	Aug. 2017	Field surveys
MED	Binhai, Tianjin	38°45' N	117°15' E	Jun. 2018	Field surveys
MED	Shijiazhuang, Hebei	38°08' N	114°33' E	Aug. 2016	Field surveys
MED	Shijiazhuang, Hebei	38°21' N	114°43' E	Jul. 2017	Field surveys
MED	Shijiazhuang, Hebei	38°00' N	114°44' E	Jul. 2017	Field surveys
MED	Shijiazhuang, Hebei	38°00' N	115°06' E	Jul. 2017	Field surveys
MED	Tangshan, Hebei	39°21' N	117°54' E	Aug. 2017	Field surveys
MED	Tangshan, Hebei	39°48' N	118°10' E	Aug. 2017	Field surveys
MED	Tangshan, Hebei	39°51' N	118°46' E	Aug. 2017	Field surveys
MED	Xingtai, Hebei	37°36' N	114°43' E	Jul. 2017	Field surveys
MED	Xingtai, Hebei	37°17' N	114°33' E	Jul. 2017	Field surveys
MED	Xingtai, Hebei	37°00' N	115°16' E	Jul. 2017	Field surveys
MED	Xingtai, Hebei	37°01' N	114°40' E	Jul. 2018	Field surveys
MED	Baoding, Hebei	39°03' N	115°35' E	Sep. 2016	Field surveys
MED	Baoding, Hebei	39°00' N	116°06' E	Sep. 2016	Field surveys
MED	Baoding, Hebei	38°56' N	115°56' E	Sep. 2016	Field surveys
MED	Baoding, Hebei	39°02' N	115°52' E	Sep. 2016	Field surveys
MED	Baoding, Hebei	38°42' N	115°47' E	Sep. 2016	Field surveys
MED	Baoding, Hebei	38°39' N	115°28' E	Aug. 2016	Field surveys
MED	Baoding, Hebei	39°10' N	115°35' E	Jul. 2017	Field surveys
MED	Baoding, Hebei	38°57' N	115°26' E	Jul. 2017	Field surveys
MED	Baoding, Hebei	38°40' N	115°08' E	Jul. 2017	Field surveys
MED	Zhangjiakou, Hebei	40°26' N	115°23' E	Jun. 2018	Field surveys
MED	Chengde, Hebei	40°53' N	117°47' E	Sep. 2016	Field surveys
MED	Chengde, Hebei	40°54' N	117°53' E	Sep. 2016	Field surveys
MED	Cangzhou, Hebei	38°06' N	117°34' E	Jun. 2018	Field surveys
MED	Handan, Hebei	36°23' N	114°21' E	Jul. 2018	Field surveys
MED	Langfang, Hebei	39°30' N	116°36' E	Jul. 2016	Field surveys
MED	Hengshui, Hebei	38°05' N	115°34' E	Aug. 2016	Field surveys
MED	Hengshui, Hebei	38°07' N	115°36' E	Aug. 2016	Field surveys
MED	Taiyuan, Shanxi	38°04' N	112°40' E	Sep. 2016	Field surveys
MED	Datong, Shanxi	40°02' N	113°17' E	Aug. 2017	Field surveys
MED	Yuncheng, Shanxi	35°43' N	111°08' E	Sep. 2017	Field surveys
MED	Xinzhou, Shanxi	39°11' N	113°15' E	Jun. 2016	Field surveys
MED	Linfen, Shanxi	36°08' N	111°26' E	Aug. 2017	Field surveys

MED	Linfen, Shanxi	36°03' N	111°35' E	Aug. 2017	Field surveys
MED	Lvliang, Shanxi	37°09' N	111°47' E	Sep. 2018	Field surveys
MED	Hohhot, Inner Mongolia	40°42' N	111°50' E	Aug. 2016	Field surveys
MED	Chifeng, Inner Mongolia	42°13' N	119°02' E	Sep. 2016	Field surveys
MED	Chifeng, Inner Mongolia	42°15' N	118°58' E	Sep. 2016	Field surveys
MED	Tongliao, Inner Mongolia	43°33' N	120°56' E	Sep. 2016	Field surveys
MED	Tongliao, Inner Mongolia	43°36' N	121°18' E	Sep. 2016	Field surveys
MED	Bayan Nur, Inner Mongolia	40°55' N	107°26' E	Aug. 2016	Field surveys
MED	Shenyang, Liaoning	41°49' N	123°33' E	Aug. 2017	Field surveys
MED	Dalian, Liaoning	39°14' N	121°43' E	Sep. 2016	Field surveys
MED	Dalian, Liaoning	39°06' N	121°42' E	Aug. 2017	Field surveys
MED	Liaoyang, Liaoning	41°16' N	123°09' E	Aug. 2016	Field surveys
MED	Changchun, Jilin	43°48' N	125°24' E	Aug. 2017	Field surveys
MED	Siping, Jilin	43°30' N	124°48' E	Aug. 2017	Field surveys
MED	Harbin, Heilongjiang	45°51' N	126°28' E	Jul. 2016	Field surveys
MED	Harbin, Heilongjiang	45°44' N	126°43' E	Aug. 2017	Field surveys
MED	Jiamusi, Heilongjiang	46°48' N	130°24' E	Aug. 2016	Field surveys
MED	Mudanjiang, Heilongjiang	44°25' N	129°30' E	Aug. 2017	Field surveys
MED	Qingpu, Shanghai	31°07' N	121°09' E	Aug. 2016	Field surveys
MED	Pudong, Shanghai	30°59' N	121°46' E	Aug. 2016	Field surveys
MED	Xuzhou, Jiangsu	34°15' N	117°23' E	Aug. 2016	Field surveys
MED	Xuzhou, Jiangsu	34°07' N	117°21' E	Aug. 2016	Field surveys
MED	Suzhou, Jiangsu	31°05' N	120°26' E	Sep. 2017	Field surveys
MED	Suzhou, Jiangsu	31°08' N	120°29' E	Sep. 2017	Field surveys
MED	Nantong, Jiangsu	31°55' N	121°08' E	Jul. 2018	Field surveys
MED	Lianyungang, Jiangsu	34°36' N	119°18' E	Aug. 2016	Field surveys
MED	Lianyungang, Jiangsu	34°46' N	119°06' E	Aug. 2016	Field surveys
MED	Huaian, Jiangsu	33°30' N	119°06' E	Aug. 2016	Field surveys
MED	Hangzhou, Zhejiang	30°12' N	120°05' E	Aug. 2016	Field surveys
MED	Hangzhou, Zhejiang	30°06' N	120°04' E	Aug. 2017	Field surveys
MED	Hangzhou, Zhejiang	30°02' N	120°11' E	Sep. 2017	Field surveys
MED	Wenzhou, Zhejiang	27°57' N	120°34' E	Aug. 2016	Field surveys
MED	Wenzhou, Zhejiang	28°04' N	120°41' E	Aug. 2016	Field surveys
MED	Wenzhou, Zhejiang	27°56' N	120°33' E	Aug. 2017	Field surveys
MED	Wenzhou, Zhejiang	28°05' N	120°30' E	Jul. 2018	Field surveys
MED	Jinhua, Zhejiang	29°05' N	119°39' E	Aug. 2016	Field surveys
MED	Jinhua, Zhejiang	29°03' N	119°45' E	Aug. 2016	Field surveys
MED	Jinhua, Zhejiang	29°02' N	119°40' E	Aug. 2017	Field surveys
MED	Jinhua, Zhejiang	29°00' N	119°38' E	Aug. 2017	Field surveys
MED	Hefei, Anhui	31°53' N	117°28' E	Aug. 2016	Field surveys
MED	Wuhu, Anhui	31°22' N	118°23' E	Aug. 2016	Field surveys
MED	Wuhu, Anhui	31°25' N	118°17' E	Sep. 2017	Field surveys
MED	Fuyang, Anhui	33°08' N	115°36' E	Jul. 2018	Field surveys
MED	Suzhou, Anhui	33°37' N	117°06' E	Aug. 2016	Field surveys

MED	Bozhou, Anhui	33°50' N	115°46' E	Sep. 2017	Field surveys
MED	Chizhou, Anhui	30°39' N	117°23' E	Aug. 2016	Field surveys
MED	Fuzhou, Fujian	25°56' N	119°14' E	Aug. 2016	Field surveys
MED	Fuzhou, Fujian	25°58' N	119°13' E	Aug. 2017	Field surveys
MED	Xiamen, Fujian	24°39' N	118°18' E	Aug. 2016	Field surveys
MED	Xiamen, Fujian	24°37' N	118°14' E	Aug. 2017	Field surveys
MED	Xiamen, Fujian	24°35' N	117°54' E	Jul. 2018	Field surveys
MED	Sanming, Fujian	26°16' N	117°38' E	Aug. 2016	Field surveys
MED	Sanming, Fujian	26°26' N	117°41' E	Aug. 2017	Field surveys
MED	Sanming, Fujian	26°29' N	117°43' E	Aug. 2017	Field surveys
MED	Zhangzhou, Fujian	24°07' N	117°36' E	Jul. 2018	Field surveys
MED	Nanchang, Jiangxi	28°43' N	115°54' E	Aug. 2016	Field surveys
MED	Pingxiang, Jiangxi	27°07' N	113°57' E	Oct. 2018	Field surveys
MED	Jiujiang, Jiangxi	29°44' N	116°07' E	Aug. 2017	Field surveys
MED	Ganzhou, Jiangxi	25°52' N	114°57' E	Aug. 2017	Field surveys
MED	Yichun, Jiangxi	28°24' N	114°47' E	Jul. 2018	Field surveys
MED	Fuzhou, Jiangxi	27°46' N	116°04' E	Oct. 2018	Field surveys
MED	Jinan, Shandong	36°41' N	117°23' E	Aug. 2016	Field surveys
MED	Qingdao, Shandong	36°12' N	120°35' E	Jul. 2018	Field surveys
MED	Zaozhuang, Shandong	34°45' N	117°25' E	Aug. 2017	Field surveys
MED	Zaozhuang, Shandong	34°53' N	117°37' E	Aug. 2017	Field surveys
MED	Dongying, Shandong	37°33' N	118°15' E	Aug. 2016	Field surveys
MED	Dongying, Shandong	37°18' N	118°28' E	Jun. 2018	Field surveys
MED	Yantai, Shandong	36°40' N	120°45' E	Jul. 2018	Field surveys
MED	Weifang, Shandong	36°54' N	118°49' E	Jun. 2018	Field surveys
MED	Taian, Shandong	35°57' N	117°15' E	Aug. 2016	Field surveys
MED	Taian, Shandong	36°12' N	116°47' E	Aug. 2016	Field surveys
MED	Linyi, Shandong	35°06' N	118°21' E	Sep. 2018	Field surveys
MED	Dezhou, Shandong	37°24' N	116°17' E	Aug. 2016	Field surveys
MED	Dezhou, Shandong	37°22' N	116°18' E	Aug. 2016	Field surveys
MED	Dezhou, Shandong	37°23' N	116°23' E	Aug. 2017	Field surveys
MED	Dezhou, Shandong	37°28' N	116°18' E	Aug. 2017	Field surveys
MED	Binzhou, Shandong	37°32' N	117°52' E	Aug. 2017	Field surveys
MED	Binzhou, Shandong	37°39' N	117°48' E	Jun. 2018	Field surveys
MED	Zhengzhou, Henan	34°55' N	113°36' E	Aug. 2016	Field surveys
MED	Luoyang, Henan	33°44' N	112°53' E	Aug. 2016	Field surveys
MED	Luoyang, Henan	33°01' N	112°25' E	Aug. 2016	Field surveys
MED	Luoyang, Henan	34°33' N	112°13' E	Sep. 2018	Field surveys
MED	Anyang, Henan	35°58' N	114°22' E	Jul. 2017	Field surveys
MED	Anyang, Henan	36°08' N	113°53' E	Jul. 2017	Field surveys
MED	Anyang, Henan	36°01' N	114°52' E	Jul. 2017	Field surveys
MED	Hebi, Henan	35°41' N	114°17' E	Jul. 2017	Field surveys
MED	Xinxiang, Henan	35°17' N	113°56' E	Aug. 2016	Field surveys
MED	Xinxiang, Henan	35°10' N	113°48' E	Aug. 2016	Field surveys

MED	Xinxiang, Henan	34°57' N	114°14' E	Jul. 2017	Field surveys
MED	Xinxiang, Henan	35°27' N	114°05' E	Jul. 2017	Field surveys
MED	Xinxiang, Henan	34°56' N	114°30' E	Jul. 2018	Field surveys
MED	Xinxiang, Henan	35°24' N	113°58' E	Jul. 2018	Field surveys
MED	Xinxiang, Henan	35°32' N	114°06' E	Jul. 2018	Field surveys
MED	Xuchang, Henan	33°53' N	111°29' E	Sep. 2018	Field surveys
MED	Nanyang, Henan	32°25' N	112°18' E	Aug. 2016	Field surveys
MED	Wuhan, Hubei	30°33' N	114°08' E	Aug. 2016	Field surveys
MED	Wuhan, Hubei	30°26' N	114°08' E	Aug. 2016	Field surveys
MED	Wuhan, Hubei	30°28' N	114°21' E	Sep. 2017	Field surveys
MED	Xiangyang, Hubei	32°05' N	112°17' E	Aug. 2016	Field surveys
MED	Xiangyang, Hubei	32°04' N	112°13' E	Sep. 2017	Field surveys
MED	Huanggang, Hubei	29°53' N	115°59' E	Aug. 2016	Field surveys
MED	Suizhou, Hubei	31°51' N	113°18' E	Aug. 2016	Field surveys
MED	Suizhou, Hubei	31°44' N	113°16' E	Aug. 2016	Field surveys
MED	Suizhou, Hubei	31°39' N	113°12' E	Sep. 2017	Field surveys
MED	Changsha, Hunan	28°10' N	113°07' E	Aug. 2016	Field surveys
MED	Changsha, Hunan	28°05' N	112°58' E	Aug. 2017	Field surveys
MED	Hengyang, Hunan	26°58' N	112°32' E	Aug. 2017	Field surveys
MED	Yueyang, Hunan	29°16' N	113°06' E	Aug. 2016	Field surveys
MED	Yueyang, Hunan	29°18' N	113°06' E	Aug. 2016	Field surveys
MED	Huaihua, Hunan	27°34' N	109°58' E	Aug. 2017	Field surveys
MED	Shantou, Guangdong	23°16' N	116°44' E	Jul. 2018	Field surveys
MED	Heyuan, Guangdong	23°58' N	114°46' E	Nov. 2017	Field surveys
MED	Nanning, Guangxi	22°51' N	108°15' E	Jun. 2016	Field surveys
MED	Liuzhou, Guangxi	24°11' N	109°25' E	Jan. 2018	Field surveys
MED	Baise, Guangxi	23°43' N	106°48' E	Mar. 2016	Field surveys
MED	Hechi, Guangxi	24°28' N	108°39' E	Jan. 2018	Field surveys
MED	Haikou, Hainan	20°00' N	110°20' E	Jul. 2016	Field surveys
MED	Sanya, Hainan	18°18' N	109°32' E	May. 2016	Field surveys
MED	Chengmai, Hainan	19°44' N	110°08' E	Aug. 2016	Field surveys
MED	Tongnan, Chongqing	30°09' N	105°49' E	Aug. 2016	Field surveys
MED	Tongnan, Chongqing	30°04' N	105°49' E	Aug. 2016	Field surveys
MED	Beibei, Chongqing	29°49' N	106°26' E	Jun. 2016	Field surveys
MED	Hechuan, Chongqing	30°03' N	106°18' E	Jun. 2016	Field surveys
MED	Chengdu, Sichuan	30°49' N	104°21' E	Aug. 2016	Field surveys
MED	Chengdu, Sichuan	30°41' N	104°18' E	Aug. 2016	Field surveys
MED	Deyang, Sichuan	31°18' N	104°21' E	Aug. 2016	Field surveys
MED	Deyang, Sichuan	31°16' N	104°21' E	Aug. 2016	Field surveys
MED	Leshan, Sichuan	29°41' N	103°38' E	Aug. 2016	Field surveys
MED	Yuxi, Yunnan	24°13' N	103°06' E	May. 2012	Hu et al. 2014
MED	Boshan, Yunnan	24°10' N	98°48' E	Oct. 2010	Hu et al. 2014
MED	Boshan, Yunnan	25°01' N	99°01' E	Oct. 2010	Hu et al. 2014
MED	Puer, Yunnan	22°47' N	101°07' E	Aug. 2012	Hu et al. 2014

MED	Lincang, Yunnan	23°30' N	99°50' E	Apr. 2018	Field surveys
MED	Chuxiong, Yunnan	25°45' N	101°45' E	Sep. 2016	Hu et al. 2018
MED	Chuxiong, Yunnan	25°44' N	101°50' E	Sep. 2016	Hu et al. 2018
MED	Chuxiong, Yunnan	25°49' N	101°44' E	Sep. 2016	Hu et al. 2018
MED	Chuxiong, Yunnan	25°51' N	101°46' E	Sep. 2016	Hu et al. 2018
MED	Chuxiong, Yunnan	25°42' N	101°53' E	Sep. 2016	Hu et al. 2018
MED	Chuxiong, Yunnan	25°52' N	101°52' E	Sep. 2016	Hu et al. 2018
MED	Chuxiong, Yunnan	25°57' N	101°52' E	Sep. 2016	Hu et al. 2018
MED	Chuxiong, Yunnan	25°55' N	101°51' E	Sep. 2016	Hu et al. 2018
MED	Chuxiong, Yunnan	25°50' N	101°50' E	Sep. 2016	Hu et al. 2018
MED	Chuxiong, Yunnan	25°46' N	101°51' E	Sep. 2016	Hu et al. 2018
MED	Chuxiong, Yunnan	25°48' N	101°49' E	Sep. 2016	Hu et al. 2018
MED	Honghe, Yunnan	23°30' N	103°24' E	Jun. 2017	Field surveys
MED	Honghe, Yunnan	24°03' N	103°23' E	May. 2014	Hu et al. 2018
MED	Honghe, Yunnan	23°30' N	103°20' E	May. 2014	Hu et al. 2018
MED	Honghe, Yunnan	23°28' N	103°19' E	Mar. 2014	Hu et al. 2018
MED	Honghe, Yunnan	23°34' N	102°26' E	June 2014	Hu et al. 2018
MED	Honghe, Yunnan	23°44' N	102°25' E	June 2014	Hu et al. 2018
MED	Honghe, Yunnan	23°40' N	103°15' E	Oct. 2014	Hu et al. 2018
MED	Honghe, Yunnan	23°44' N	103°15' E	Mar. 2014	Hu et al. 2018
MED	Honghe, Yunnan	23°43' N	105°15' E	Mar. 2014	Hu et al. 2018
MED	Honghe, Yunnan	22°38' N	103°57' E	Oct. 2014	Hu et al. 2018
MED	Honghe, Yunnan	22°32' N	103°57' E	Oct. 2014	Hu et al. 2018
MED	Honghe, Yunnan	23°29' N	103°24' E	Sep. 2011	Hu et al. 2014
MED	Honghe, Yunnan	23°46' N	103°15' E	May. 2012	Hu et al. 2014
MED	Honghe, Yunnan	24°06' N	103°23' E	May. 2012	Hu et al. 2014
MED	Xishuangbanna, Yunnan	21°27' N	101°34' E	Aug. 2016	Field surveys
MED	Xishuangbanna, Yunnan	21°40' N	100°43' E	Apr. 2018	Field surveys
MED	Xishuangbanna, Yunnan	21°32' N	100°40' E	Apr. 2018	Field surveys
MED	Xishuangbanna, Yunnan	21°56' N	100°45' E	Sep. 2016	Hu et al. 2018
MED	Xishuangbanna, Yunnan	21°30' N	101°33' E	Aug. 2012	Hu et al. 2014
MED	Xishuangbanna, Yunnan	21°59' N	100°16' E	Aug. 2012	Hu et al. 2014
MED	Xishuangbanna, Yunnan	22°24' N	101°23' E	Aug. 2012	Hu et al. 2014
MED	Dehong, Yunnan	24°01' N	97°51' E	Apr. 2018	Field surveys
MED	Xi'an, Shaanxi	34°10' N	109°08' E	Sep. 2016	Field surveys
MED	Xi'an, Shaanxi	34°06' N	108°29' E	Aug. 2017	Field surveys
MED	Baoji, Shaanxi	34°21' N	107°27' E	Aug. 2016	Field surveys
MED	Baoji, Shaanxi	34°23' N	107°23' E	Aug. 2017	Field surveys
MED	Yan'an, Shaanxi	36°52' N	109°20' E	Aug. 2016	Field surveys
MED	Yan'an, Shaanxi	36°55' N	109°23' E	Aug. 2017	Field surveys
MED	Hanzhong, Shaanxi	33°05' N	107°09' E	Aug. 2016	Field surveys
MED	Yulin, Shaanxi	37°59' N	109°51' E	Aug. 2016	Field surveys
MED	Yulin, Shaanxi	38°18' N	109°33' E	Aug. 2017	Field surveys
MED	Yulin, Shaanxi	38°18' N	109°38' E	Aug. 2017	Field surveys

MED	Lanzhou, Gansu	35°51' N	104°08' E	Sep. 2016	Field surveys
MED	Lanzhou, Gansu	35°50' N	104°07' E	Sep. 2016	Field surveys
MED	Lanzhou, Gansu	36°06' N	103°41' E	Aug. 2018	Field surveys
MED	Lanzhou, Gansu	36°07' N	103°38' E	Aug. 2018	Field surveys
MED	Tianshui, Gansu	34°42' N	105°40' E	Aug. 2016	Field surveys
MED	Tianshui, Gansu	34°31' N	106°03' E	Aug. 2016	Field surveys
MED	Zhangye, Gansu	39°03' N	100°08' E	Aug. 2016	Field surveys
MED	Zhangye, Gansu	38°55' N	100°27' E	Aug. 2018	Field surveys
MED	Jiuquan, Gansu	39°45' N	98°32' E	Aug. 2016	Field surveys
MED	Jiuquan, Gansu	40°29' N	95°49' E	Jul. 2017	Field surveys
MED	Jiuquan, Gansu	40°31' N	95°46' E	Aug. 2018	Field surveys
MED	Yinchuan, Ningxia	38°28' N	106°22' E	Sep. 2016	Field surveys
MED	Guyuan, Ningxia	36°03' N	106°17' E	Sep. 2016	Field surveys
MED	Zhongwei, Ningxia	37°31' N	105°16' E	Sep. 2016	Field surveys
MED	Urumqi, Xinjiang	44°01' N	87°26' E	Aug. 2016	Field surveys
MED	Urumqi, Xinjiang	43°48' N	87°34' E	May. 2016	Jia et al. 2021
MED	Urumqi, Xinjiang	45°29' N	84°57' E	May. 2016	Jia et al. 2021
MED	Tulufan, Xinjiang	42°40' N	89°15' E	Jul. 2016	Field surveys
MED	Tulufan, Xinjiang	42°54' N	89°29' E	Jul. 2016	Field surveys
MED	Tulufan, Xinjiang	42°57' N	89°11' E	Aug. 2016	Field surveys
MED	Tulufan, Xinjiang	42°52' N	89°19' E	Jul. 2017	Field surveys
MED	Tulufan, Xinjiang	42°59' N	89°09' E	Jun. 2015	Jia et al. 2021
MED	Tulufan, Xinjiang	42°57' N	89°06' E	Jun. 2015	Jia et al. 2021
MED	Tulufan, Xinjiang	42°48' N	88°27' E	Jun. 2015	Jia et al. 2021
MED	Tulufan, Xinjiang	42°46' N	88°41' E	Apr. 2016	Jia et al. 2021
MED	Tulufan, Xinjiang	42°49' N	88°38' E	Apr. 2016	Jia et al. 2021
MED	Tulufan, Xinjiang	42°46' N	89°33' E	Apr. 2016	Jia et al. 2021
MED	Tulufan, Xinjiang	42°47' N	89°43' E	Apr. 2016	Jia et al. 2021
MED	Tulufan, Xinjiang	42°43' N	89°41' E	Apr. 2016	Jia et al. 2021
MED	Hami, Xinjiang	42°53' N	93°27' E	Aug. 2016	Field surveys
MED	Hami, Xinjiang	42°50' N	93°28' E	Aug. 2016	Field surveys
MED	Hami, Xinjiang	43°00' N	93°35' E	Apr. 2016	Jia et al. 2021
MED	Changji, Xinjiang	44°00' N	87°18' E	May. 2016	Jia et al. 2021
MED	Changji, Xinjiang	44°09' N	88°01' E	Mar. 2017	Jia et al. 2021
MED	Bortala, Xinjiang	45°10' N	82°33' E	Sep. 2016	Jia et al. 2021
MED	Bayingol, Xinjiang	41°46' N	86°08' E	Mar. 2016	Jia et al. 2021
MED	Kashi, Xinjiang	37°40' N	76°13' E	Jun. 2018	Field surveys
MED	Kashi, Xinjiang	38°24' N	77°17' E	Sep. 2015	Jia et al. 2021
MED	Kashi, Xinjiang	39°24' N	75°54' E	Jul. 2015	Jia et al. 2021
MED	Kashi, Xinjiang	38°21' N	77°10' E	Aug. 2016	Jia et al. 2021
MED	Kashi, Xinjiang	38°17' N	77°11' E	Aug. 2016	Jia et al. 2021
MED	Kashi, Xinjiang	39°24' N	76°08' E	Sep. 2015	Jia et al. 2021
MED	Kashi, Xinjiang	39°14' N	76°22' E	Sep. 2015	Jia et al. 2021
MED	Kashi, Xinjiang	38°18' N	77°56' E	Apr. 2016	Jia et al. 2021

MED	Hetian, Xinjiang	37°14' N	79°41' E	Aug. 2016	Jia et al. 2021
MED	Yili, Xinjiang	44°14' N	80°51' E	Aug. 2018	Field surveys
MED	Yili, Xinjiang	44°11' N	80°39' E	Mar. 2018	Field surveys
MED	Yili, Xinjiang	43°51' N	81°25' E	May. 2015	Jia et al. 2021
MED	Shihezi, Xinjiang	44°18' N	86°04' E	May. 2016	Jia et al. 2021
MED	Alaer, Xinjiang	40°32' N	81°16' E	Mar. 2017	Jia et al. 2021

[1] References cited in Table S1:

Hu, J.; Jiang, Z.L.; Nardi, F.; Liu, Y.Y.; Luo, X.R.; Li, H.X.; Zhang, Z.K. Members of *Bemisia tabaci* (Hemiptera: Aleyrodidae) Cryptic Species and the Status of Two Invasive Alien Species in the Yunnan Province (China). *Journal of Insect Science* 2014, 14, 1-8.

Hu, J.; Zhang, X.; Jiang, Z.; Zhang, F.; Liu, Y.; Li, Z.; Zhang, Z. New putative cryptic species detection and genetic network analysis of *Bemisia tabaci* (Hempitera: Aleyrodidae) in China based on mitochondrial COI sequences. *Mitochondrial DNA A DNA Mapp Seq Anal* 2018, 29, 474-484.

Jia, Z.Z.; Fu, K.Y.; Guo, W.C.; Jiang, W.H.; Ahmat, T.; Ding, X.H.; He, J.; Wang, X.W. CAP Analysis of the Distribution of the Introduced *Bemisia tabaci* (Hemiptera: Aleyrodidae) Species Complex in Xinjiang, China and the Southerly Expansion of the Mediterranean Species. J*ournal of Insect Science* 2021, 21, 14.

Table S2. The correlation matrix of environmental variables.

	bio2	bio3	bio4	bio5	bio6	bio7	bio8	bio9	bio10	bio11	bio12	bio13	bio14	bio15	bio16	bio17	bio18	bio19	elev
**bio1**	−0.59	−0.15	−0.31	0.81	0.91	−0.43	0.84	0.88	0.89	0.91	0.64	0.57	0.62	−0.53	0.59	0.62	0.55	0.59	−0.72
**bio2**		0.29	0.47	−0.23	−0.74	0.67	−0.33	−0.57	−0.40	−0.67	−0.85	−0.80	−0.76	0.46	−0.81	−0.75	−0.78	−0.71	0.40
**bio3**			−0.67	−0.50	0.10	−0.49	−0.49	0.21	−0.49	0.16	−0.05	−0.08	−0.18	0.09	−0.03	−0.16	−0.01	−0.12	0.68
**bio4**				0.29	−0.66	0.97	0.22	−0.64	0.16	−0.67	−0.56	−0.46	−0.43	0.30	−0.51	−0.44	−0.50	−0.45	−0.37
**bio5**					0.50	0.18	0.96	0.50	0.98	0.51	0.24	0.21	0.33	−0.36	0.20	0.32	0.16	0.29	−0.90
**bio6**						−0.76	0.56	0.95	0.63	0.99	0.77	0.68	0.72	−0.56	0.71	0.72	0.67	0.69	−0.42
**bio7**							0.08	−0.71	0.02	−0.75	−0.70	−0.61	−0.57	0.37	−0.66	−0.57	−0.64	−0.57	−0.20
**bio8**								0.52	0.97	0.56	0.33	0.33	0.33	−0.29	0.31	0.32	0.31	0.26	−0.91
**bio9**									0.60	0.97	0.66	0.55	0.65	−0.58	0.59	0.65	0.54	0.66	−0.35
**bio10**										0.63	0.40	0.38	0.44	−0.40	0.36	0.44	0.33	0.40	−0.92
**bio11**											0.74	0.65	0.68	−0.54	0.68	0.68	0.64	0.66	−0.40
**bio12**												0.95	0.86	−0.43	0.97	0.86	0.94	0.84	−0.38
**bio13**													0.73	−0.21	0.99	0.73	0.98	0.70	−0.41
**bio14**														−0.60	0.75	0.99	0.66	0.97	−0.37
**bio15**															−0.27	−0.61	−0.20	−0.60	0.27
**bio16**																0.75	0.98	0.73	−0.38
**bio17**																	0.66	0.98	−0.37
**bio18**																		0.63	−0.36
**bio19**																			−0.32

Table S3. Potential suitable areas for MEAM1 and MED of the *Bemisia tabaci* species complex in China under current and future climate scenarios.

Cryptic species	Climate scenarios and periods		Area (10^4 km^2)			
			Slightly suitable	Moderately suitable	Highly suitable	Total
MEAM1	Current		101.66	60.84	26.18	188.67
	SSP1-2.6	2030s	88.09	72.96	70.83	231.88
		2050s	100.51	79.61	89.08	269.19
		2070s	103.88	69.76	52.76	226.40
		2090s	100.40	78.40	75.31	254.11
	SSP2-4.5	2030s	109.98	84.24	64.35	258.57
		2050s	109.09	86.24	106.33	301.66
		2070s	105.99	92.41	129.73	328.14
		2090s	103.41	92.45	133.86	329.73
	SSP3-7.0	2030s	100.84	76.13	60.16	237.14
		2050s	118.74	86.16	100.35	305.25
		2070s	99.96	86.15	152.35	338.46
		2090s	86.05	85.47	179.52	351.05
	SSP5-8.5	2030s	109.14	80.87	83.24	273.26
		2050s	100.92	86.23	115.05	302.21
		2070s	97.92	84.72	145.88	328.52
		2090s	89.82	86.62	176.68	353.13
MED	Current		44.67	58.27	51.05	153.99
	SSP1-2.6	2030s	57.78	62.01	87.90	207.70
		2050s	51.27	76.23	109.38	236.87
		2070s	55.98	63.56	77.46	197.00
		2090s	58.97	58.05	86.55	203.58
	SSP2-4.5	2030s	60.85	62.93	84.95	208.72
		2050s	65.90	66.22	102.01	234.13
		2070s	73.58	82.73	118.86	275.18
		2090s	81.13	93.72	115.99	290.84
	SSP3-7.0	2030s	58.23	61.09	84.44	203.77
		2050s	66.38	79.59	107.97	253.94
		2070s	77.43	86.32	135.25	299.00
		2090s	78.41	100.15	155.41	333.96
	SSP5-8.5	2030s	58.18	63.41	85.04	206.63
		2050s	71.68	80.25	110.20	262.13
		2070s	74.94	79.69	130.54	285.17
		2090s	75.95	94.45	159.38	329.77

SSP, shared socioeconomic pathway.

Table S4. The geographic coordinates of the distributional centroid of MEAM1 and MED cryptic species of the *Bemisia tabaci* species complex in China under current and future climate scenarios and their migration distance relative to the previous period.

Cryptic species	Climate scenarios and periods		Longitude	Latitude	Distance (km)
MEAM1	Current		103°49'E	33°27'N	—
	SSP1-2.6	2030s	105°02'E	32°46'N	136.46
		2050s	104°13'E	32°59'N	80.51
		2070s	103°20'E	33°38'N	109.62
		2090s	104°06'E	33°49'N	74.24
	SSP2-4.5	2030s	103°22'E	33°45'N	53.75
		2050s	102°47'E	34°36'N	107.62
		2070s	103°15'E	34°15'N	57.57
		2090s	102°36'E	34°34'N	70.86
	SSP3-7.0	2030s	103°19'E	33°34'N	48.23
		2050s	103°05'E	34°00'N	52.77
		2070s	103°10'E	34°40'N	74.27
		2090s	102°55'E	34°34'N	25.14
	SSP5-8.5	2030s	103°13'E	34°20'N	112.33
		2050s	103°12'E	34°11'N	15.84
		2070s	103°10'E	34°18'N	13.98
		2090s	102°53'E	34°11'N	29.47
MED	Current		109°01'E	33°08'N	—
	SSP1-2.6	2030s	108°60'E	32°36'N	59.30
		2050s	108°01'E	32°30'N	93.54
		2070s	108°06'E	33°33'N	117.20
		2090s	108°18'E	34°11'N	73.07
	SSP2-4.5	2030s	107°43'E	33°18'N	123.38
		2050s	106°13'E	34°27'N	188.02
		2070s	107°16'E	34°10'N	102.04
		2090s	106°25'E	34°49'N	105.80
	SSP3-7.0	2030s	108°03'E	33°11'N	89.62
		2050s	107°02'E	33°20'N	97.21
		2070s	106°55'E	34°56'N	179.34
		2090s	106°45'E	35°26'N	56.51
	SSP5-8.5	2030s	107°09'E	33°56'N	194.73
		2050s	107°38'E	34°12'N	53.65
		2070s	107°08'E	35°04'N	106.10
		2090s	107°01'E	35°02'N	10.93

SSP, shared socioeconomic pathway.

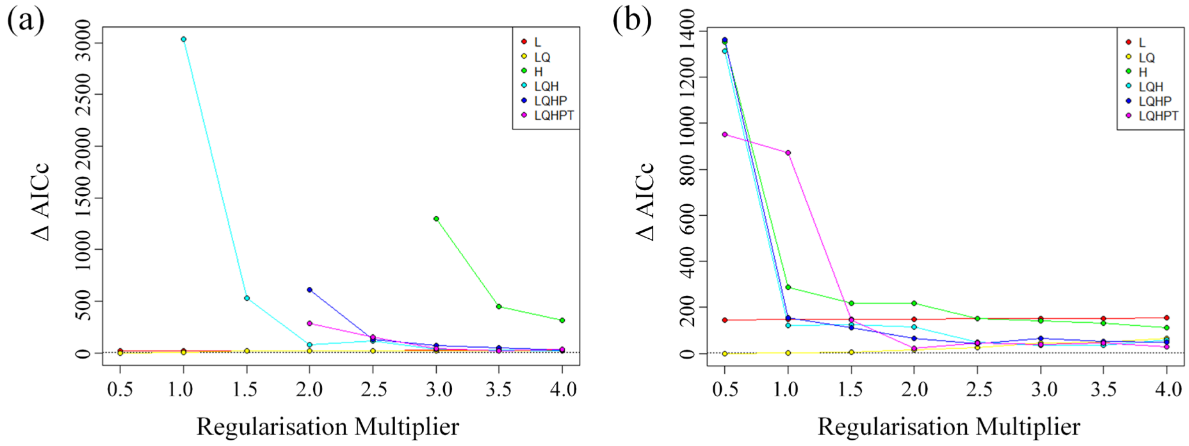

Figure S1. ENMeval output for (a) MEAM1 and (b) MED cryptic species of the *Bemisia tabaci* species complex, showing that the optimal parameter combination of feature class and regularization multiplier in the MaxEnt models was: LQ and 0.5, respectively, for both of them. AICc, Akaike information criterion coefficient.

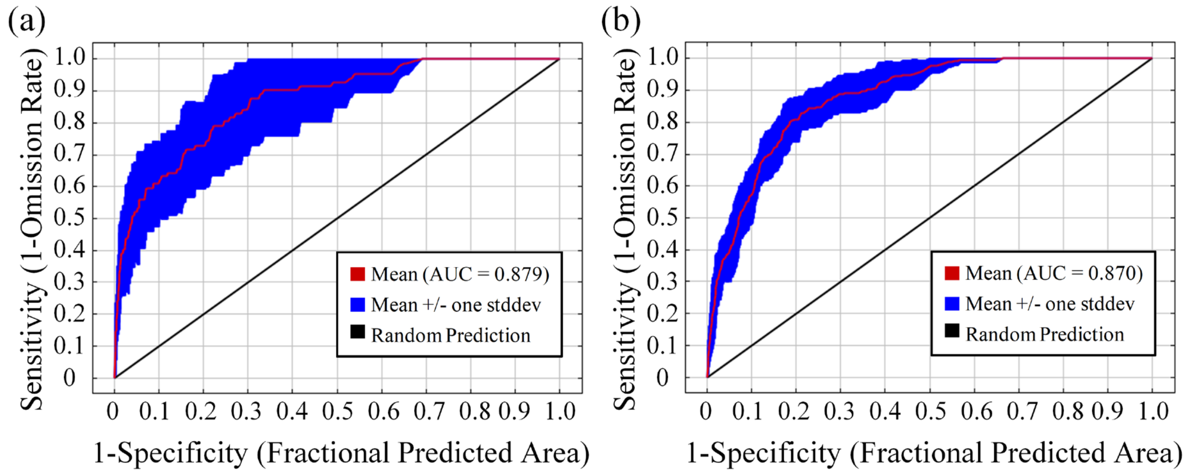

Figure S2. Receiver operating characteristic curve verification of the prediction for (a) MEAM1 and (b) MED cryptic species of the *Bemisia tabaci* species complex. AUC, area under the curve.
